# Evaluation of the Effectiveness of Buprenorphine-Naloxone on Opioid Overdose and Death among Insured Patients with Opioid Use Disorder in the United States

**DOI:** 10.3390/pharma1030010

**Published:** 2022-11-24

**Authors:** Tianyu Sun, Natallia Katenka, Stephen Kogut, Jeffrey Bratberg, Josiah Rich, Ashley Buchanan

**Affiliations:** 1 Department of Pharmacy Practice, College of Pharmacy, University of Rhode Island, Kingston, RI 02881, USA; 2 Department of Computer Science and Statistics, College of Art and Science, University of Rhode Island, Kingston, RI 02881, USA; 3 Department of Epidemiology, Brown University School of Public Health, Providence, RI 02903, USA; 4 The Warren Alpert Medical School of Brown University, Providence, RI 02903, USA

**Keywords:** buprenorphine naloxone, causal inference, marginal structural models, opioid overdose, administrative claims

## Abstract

Opioid use disorder (OUD) is a chronic disease requiring long-term treatment and is associated with opioid overdose and increased risk of mortality. However, existing randomized clinical trials focused on short-term treatment engagement and detoxification rather than overdose or mortality risk due to limited follow-up time and ethical considerations. We used a hypothetical trial framework to conduct a retrospective cohort study to assess the effectiveness of time-varying buprenorphine-naloxone on opioid overdose and death. We identified 58,835 insured adult patients with OUD diagnosis in the US, 2010–2017. We fit a marginal structural model using inverse probability weighting methods to account for measured baseline and time-varying confounders, as well as selection bias due to possibly differential loss-to-follow-up. We found that receipt of buprenorphine-naloxone was associated with reduced risk of opioid overdose (hazard ratio (HR) = 0.66, 95% confidence interval (CI): 0.49, 0.91), death (HR = 0.24, 95% CI: 0.08, 0.75), and overdose or death (HR = 0.58, 95% CI: 0.40, 0.84). The E-value for death was 7.8, which was larger than the upper 95% CI of the association between each measured baseline variable and all-cause death, which implies that the unmeasured confounding itself may not explain away the estimated effect of treatment on the endpoint of all-cause mortality.

## 1. Introduction

The opioid overdose crisis is a major concern in the United States, exacerbated by the COVID-19 pandemic, which further limited treatment access among patients with opioid use disorder and chronic pain [1]. Furthermore, the COVID-19 global pandemic accelerated the increasing drug overdose deaths. According to the National Institute on Drug Abuse, in 2019 alone, there were 70,630 drug poisoning deaths. This number dramatically ramped up to 91,799 in 2020, the highest year-to-year increment was 2019 to 2020 May, which was 46% [1,2]. This syndemic disproportionately affects minoritized populations, resulting in widening of health disparities. [3]. Efforts had been made to address this public health issue. The US Food and Drug Administration approved buprenorphine products (with/without naloxone) as medication for opioid use disorder [4,5]. The American Society of Addiction Medicine provided a national practice guideline suggesting prescribing buprenorphine for patients with opioid use disorder (OUD) and the Journal of the American Medical Association published a clinical guideline for the management of OUD specifically stated that the buprenorphine-naloxone (BUP-NX) product was the recommended treatment [6–8].

The benefit of BUP-NX treatment for patients with opioid use disorder is supported by evidence from clinical trials. One randomized clinical trial compared BUP-NX versus clonidine on retention and treatment success (negative urine sample test of illicit opioids) at the end of a 14-day-follow-up [9]. The result showed that there were significantly more patients who achieved treatment retention and success among the BUP-NX arm than the clonidine arm in both inpatient and outpatient settings. Another randomized clinical trial compared the timely BUP-NX initiation by emergency department versus brief negotiation and/or referral on treatment retention, self-reported illegal drug use, and urine test of illegal drug after 30-day follow-up [10]. This study demonstrated that patients diagnosed with OUD who visited the emergency department and initiated BUP-NX had significantly higher treatment engagement and lower illicit drug use than patients who only received brief negotiation and/or referral.

## 2. Study Rationale and Objective

Randomized clinical trials thus far have provided evidence supporting the short-term efficacy of BUP-NX on detoxification. However, opioid use disorder is a chronic disease requiring long-term treatment and is associated with opioid overdose and increased risk of mortality [11,12]. The American Society of Addiction Medicine suggested that the buprenorphine treatment discontinuation should be a slow process with ‘indefinite duration’ [6]. Martin et al. also stated that buprenorphine should be prescribed as long as it still could benefit the patient [13]. Given the longer trajectory of this chronic disease and health-related outcomes, previously conducted clinical trials did not provide information about the real-world effectiveness of this medication for a longer duration. However, it is currently not ethical to conduct a randomized trial to directly assess the efficacy of BUP-NX (comparing to placebo) due to a lack of clinical equipoise.

When a randomized trial is not feasible or ethical, conducting a retrospective cohort study using routinely-collected data to estimate the effectiveness of the treatment is an alternative. In the light of the real-world evidence, Hernán et al. developed a framework to improve the design and conduct of retrospective studies [14]. This framework provides guidance and methods to leverage the routinely-collected data to approximate the key features of the hypothetical randomized trial. There is published evidence that such an approach is achievable by clearly addressing and stating known limitations of the observational data and analytic approach [14,15]. Given the lack of randomized trials with long-term follow-up, we aim to estimate the effectiveness of buprenorphine-naloxone (BUP-NX) on health outcomes, specifically opioid overdose and all-cause mortality, using large, routinely-collected observational health data sets with the design informed by a hypothetical randomized trial.

## 3. Materials and Methods

We first conceptualized a hypothetical trial to address the research question, regardless its feasibility in the real world. Then, we designed a retrospective cohort study to emulate the hypothetical trial as closely as possible using an administrative claims data: the Optum’s de-identified Clinformatics ^®^ Data Mart (OptumInsight, Eden Prairie, MN, USA) from 1 January 2010 to 31 December 2017. Major protocol elements of the hypothetical randomized trial and the rationale, design, and method for the retrospective cohort study emulating the conceptual trial are provided in Table 1. The target population included insured adults diagnosed with OUD in the US. We identified patients who had at least two outpatient claims or one inpatient claim of OUD as the primary or secondary diagnosis. The diagnosis was identified by the International Classification of Disease 9/10th revision, clinical modification (ICD-9/10-CM) code (Supplemental Table S1).

**Figure 1.**
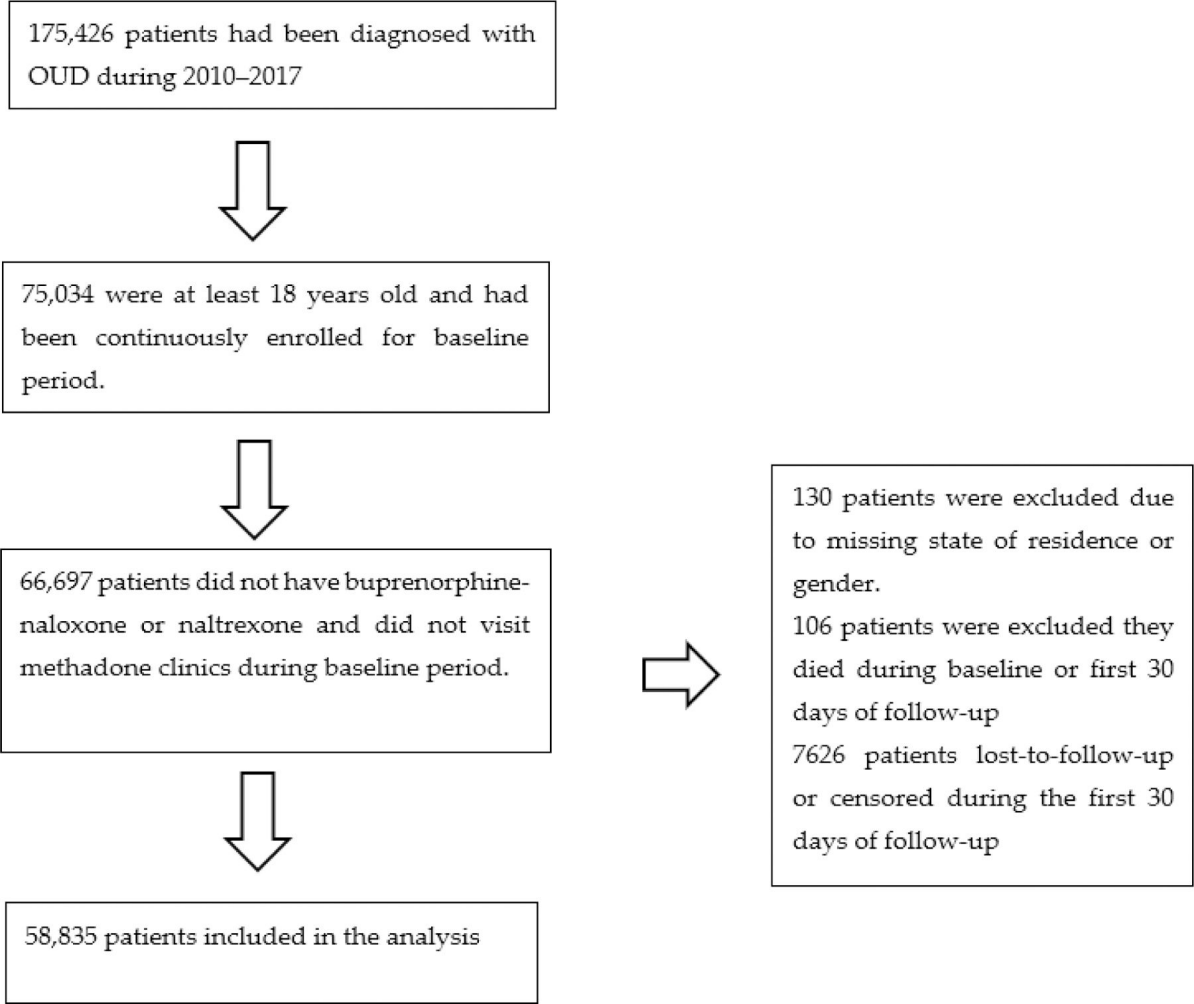
Flowchart of analytical sample building for retrospective cohort study of insured patients with opioid use disorder in the United States, 2010–2017.

The first eligible OUD diagnosis in the database was defined as the start of study follow-up (time zero) for that patient. The one-year period before time zero was defined as the baseline period. Patients were followed for up to two years after their OUD diagnosis. The follow-up ended when the outcome occurred, the patient was lost-to-follow-up, or the patient reached the end of the database (31 December 2017), whichever happened first. All patients were required to be continuously enrolled in the database during the baseline period and at least 18 years old at time zero with at least 30 days of follow-up after baseline to allow for ascertainment of outcomes. Patients who had received buprenorphine-naloxone or naltrexone, or who had visited methadone clinics during the baseline period were excluded. Patients who died during the baseline period were also excluded. Patients missing their state of residence or gender information were excluded (Figure 1). No imputation method was applied because the proportion of missing data was negligible (0.22%). Loss to follow-up was defined as: (1) the patient lost insurance coverage; or (2) the patient did not have any claims (medical, confinement, or pharmacy) for 6 months. In both cases, they were considered as lost-to-follow-up at their last encounter [16].

Several existing studies have shown that patients are at a high risk of overdose and death during the first four-week after treatment cessation [17,18] and in light of this, previous retrospective cohort study selected a monthly frequency to update time-varying exposures [19]. The exposure of interest was the time-varying status of receiving any BUP-NX dispensing in each 30-day interval during the follow-up period. The exposure was identified from pharmacy claims by brand or generic names, which contain both buprenorphine and naloxone. The exposure status was updated every 30 days based on the date of dispensing and days of supply. The study outcomes were opioid overdose, all-cause death, and a composite endpoint of both (whichever occurred first). The opioid overdose event was identified by the ICD-9/10-CM code from both inpatient/outpatient claims (Supplemental Table S1). The overdose outcome included both non-fatal and fatal overdose that received treatment at the hospital. Mortality information was originated from the Social Security Administrative Death Master File. Only year and month of death were available in this deidentified claims database.

We identified the following covariates that were known or suspected risk factors for the outcomes: age, gender, type of insurance, region, type of insurance product, history of opioid overdose, chronic pain, depression, alcohol use disorder, opioid use, and Charlson comorbidity index (CCI) (Supplemental Table S1) [20–22]. The CCI score included 17 major comorbidities [23]. This score is widely used to weigh and quantify the burden of cohort comorbidities and a strong predictor for mortality [24]. To adjust for time-varying confounding, the following variables were derived and updated every 30 days: use of benzodiazepine (yes/no), use of naltrexone products (yes/no), use of anti-depressants (yes/no), visiting methadone clinic (yes/no), alcohol use disorder (yes/no), and daily morphine milligram equivalence (MME) dose. The daily MME is a standard measure of the total dosage including different opioids, such as morphine, oxycodone, mono-ingredient buprenorphine, etc. The conversion factor and formula were provided by the Centers for Disease Control and Prevention to standardize different types of opioids into comparable dosages and to calculate the average daily MME of each dispensing [25].

Patient baseline characteristics were summarized overall at baseline. Descriptive statistics were calculated as means with standard deviations (SD) for continuous variables and percentages for categorical variables. The outcomes were analyzed with time-to-event statistical methodology and relative comparisons were estimated as hazard ratios (HR). We first fit an unadjusted Cox model with the BUP-NX exposure and time-to-event outcomes. The exposure was parameterized as a time-varying indicator of receipt of any BUP-NX in the 30-day interval [26,27]. We fit a marginal structural model (MSM) using inverse probability weights to estimate the association between time-varying BUP-NX dispensing on three outcomes: (1) opioid overdose; (2) death; (3) opioid overdose or death among patients with OUD. If the identification assumptions (i.e., well-defined interventions, conditional exchangeability, and positivity) are met, the estimated effect from this model can be interpreted as a causal hazard ratio comparing the hazard of opioid overdose under receipt of BUP-NX versus no receipt of BUP-NX during follow-up [15,28]. This contrast could be interpreted as a conceptual randomized trial in which participants are re-randomized at each monthly time interval [29]. We employed inverse probability of treatment weights to adjust for measured time-varying covariates to control for confounding as well as the treatment history and also included an inverse probability of censoring weights to address possible selection bias due to possibly differential loss to follow-up [30,31]. Essentially, this weighting approach creates a pseudo-population in which the exposure is not associated with the confounders and no one was lost to follow-up [15,32]. The stabilized treatment and censoring weights were estimated by two separate pooled logistic regression models, respectively. Baseline variables, time-varying variables, and treatment history were included in estimation of the denominator of treatment weights. Only baseline variables were included to estimate the numerator of treatment weights. Treatment was included as a variable in the model to estimate the censoring weights [32]. Using a stabilized weight in the outcome model may be more efficient in this setting [15,28]. We fit a flexible function of time (measured in months since OUD diagnosis) using cubic splines with four knots and this was included in each of the weight models [32,33]. Due to the use of stabilized weights, the same set of baseline variables was also included in the outcome model [32]. We used robust standard errors for parameter estimates to obtain Wald-type confidence intervals [30,32]. We also used the inverse probability weighted Cox model to generate adjusted cumulative incidence curves.

We designed a series sensitivity analyses to assess the model fit, robustness of the results regarding study design, and vulnerability to potential unmeasured confounding. We checked the exposure and censoring weight distributions. Then, we evaluated an alternative loss-to-follow-up definition established by Lesko et al. [16]. This alternative definition may be more appropriate when outcome information is obtained from an external source, such as a death registry. After the last encounter of patients, we carried forward their information for six additional 30-day intervals and calculated the corresponding hazard ratios (HRs). To further quantify the robustness of the results to the no unmeasured confounding assumption, we calculated the E-values and compared with the association between the outcome and known risk factors [34–36]. All statistical analysis was done by SAS 9.4 (SAS Institute, Cary, NC, USA) and all statistical tests were two-sided and conducted at the 0.05 significance level.

## 4. Results

We successfully identified 58,835 adult patients diagnosed with opioid use disorder from 2010 to 2017. Approximately half of the patients were male (49%) and the mean age of the analytical sample was 51 years (SD = 17.6). Among all patients, 55% were enrolled in commercial insurance and the rest were enrolled in Medicare Advantage. Most patients used a health maintenance organization or point of service as their insurance product (69%). During the one-year baseline period, the overall disease burden measured by the CCI was 1 (SD = 1.64), which means that on average patients had one out of the seventy comorbidities included in CCI. During the baseline period, a small proportion of patients had a documented history of opioid overdose (4%) and 15% had documented alcohol use disorder. Almost half of the patients had depression (47%) and more than two-thirds of patients had chronic pain conditions (79%) during the baseline period (Table 2).

The total person-time for opioid overdose, death, and composite endpoint were 63,910 person-years, 65,180 person-years, and 63,881 person-years, respectively. During the study follow-up out of a total of 58,835 patients, 1908 (3.2%) patients had at least one opioid overdose, 1051 (1.8%) patients died, and 2874 (4.9%) patients experienced the composite endpoint of either death or opioid overdose during the follow-up. There were 5722 patients who had received at least one BUP-NX dispensing before having an opioid overdose, or were lost to follow-up (whichever came first). For the other two endpoints (death and composite), there were 5835 and 5720 patients who had at least one BUP-NX dispensing before overdose, death, or were lost to follow-up (whichever came first). We focused on discussion on results from the MSM that accounted for both measured baseline and time-varying confounding and possibly differential loss to follow-up (Table 3). Patients who received BUP-NX during follow-up had a 34% lower hazard of overdose (HR = 0.66, 95% confidence interval (CI): 0.49, 0.91), a 76% lower hazard of death (HR = 0.24, 95% CI: 0.08, 0.75), and a 42% lower hazard of the composite endpoint (HR = 0.58, 95% CI: 0.40, 0.84) during follow-up compared to patients who were not on BUP-NX.

The mean of the product of the two weights (i.e., treatment weights and drop-out weights) was close to 1, which suggests no evidence of model misspecification or positivity assumption violations (Table 3) [15]. The weighted cumulative incidence curves for each of the three endpoints are provided in Supplemental Figures S1–S3 [37]. Patients who did not receive BUP-NX had a higher estimated cumulative incidence of opioid overdose or death than patients who received BUP-NX. Under different loss-to-follow-up definition in the sensitivity analysis, the average follow-up time was longer and more events were observed for all three endpoints in the sensitivity analysis than the main analysis (Table 3 and Supplemental Table S2). The HRs estimated for opioid overdose, death, and composite endpoint were 0.73 (95% CI: 0.55, 0.96), 0.45 (95% CI: 0.22, 0.92), and 0.69 (95% CI: 0.53, 0.90). The point estimators were similar to the main analysis, but slightly attenuated towards the null, likely due to longer follow-up with few additional events. However, their corresponding 95% confidence intervals were comparable to the main analysis. The E value for estimated HRs of three outcomes (opioid overdose, death, and composite endpoint) were 2.4, 7.8, and 2.8, respectively (Table 3). Particularly for the outcome of death, the E-value is much larger than the upper 95% CI for any measured baseline variables included in the model (Supplemental Table S3).

## 5. Discussion

In this retrospective cohort study, we evaluated the effectiveness of time-varying buprenorphine-naloxone dispensing on reducing subsequent opioid overdose and/or death through an application of causal inference methods with a trial emulation framework using routinely-collected health data. The data source is a large national administrative claims database from 2010 to 2017 and included 58,835 patients diagnosed with OUD in the United States. A marginal structural model was applied to analyze the time-to-event outcomes and conduct inference in an observational setting. With adjustment for measured baseline and time-varying variables and possible selection bias due to loss to follow-up, we found that receiving BUP-NX during the two-year follow-up could decrease the hazard of opioid overdose and/or death among patients who were diagnosed with OUD, as compared to not receiving BUP-NX during follow-up.

Medication treatment retention or sustained medication treatment has been shown to be one of the most important protective factors associated with a lower risk of opioid overdose. Furthermore, the risk of overdose after treatment discontinuation is high regardless of the treatment duration before discontinuation [38]. Chang et al. used the Maryland prescription drug monitoring program (2015 to 2016) to investigate protective and harmful risk factors for nonfatal opioid overdose. Their results demonstrated that an additional 100-day buprenorphine treatment was associated with a 36% reduction in risk of opioid overdose among patients with opioid use disorder and received at least one buprenorphine dispensing (odds ratio = 0.64, 95% CI: 0.60, 0.69) [39]. Another study conducted by Morgan et al. used a Cox model with a time-varying exposure and adjusted for baseline confounding found that receiving buprenorphine dispensing was associated with a lower hazard of opioid overdose (adjusted HR = 0.40, 95% CI: 0.35, 0.46), compared to not receiving treatment [40]. Larochelle et al. focused on adult patients, who had already experienced a nonfatal opioid overdose, used a Cox regression model with time-varying exposure and confounding variables, and found that buprenorphine treatment was associated with lower all-cause mortality (adjusted HR = 0.63, 95% CI: 0.46, 0.87) and lower opioid-related mortality (adjusted HR = 0.62, 95% CI: 0.41, 0.92), compared to no medication treatment [19].

Our study provides new insights into OUD treatment by considering important improvements in study design and statistical methodology. Conditioning on treatment initiation leads to a depletion of potential patients who comprise an important part of the target population of all insured adult patients diagnosed with OUD. This could distort the study sample from the target patient population of interest, rendering questions among the entire patient population with OUD difficult to address. Consequently, the finding otherwise would be limited to patients who had both the disease and had received a certain type and duration of treatment. The eligibility criteria to identify the target patient population and definition of the start of follow-up in this study were carefully designed based on the concept of trial emulation for an ideal randomized study to address this research question [14]. In a real-world setting, patients with OUD who sought care are not guaranteed to receive pharmacological treatment after their diagnosis [41,42]. Taking this into consideration, instead of conditioning on medication treatment initiation, we included all insured adult patients who had been diagnosed with OUD regardless of initiation status. Thus, our study population may better approximate the target population of the insured adult patients with OUD whom physicians would likely encounter in clinical practice.

From a statistical method perspective, the time-dependent Cox model can be subject to bias when the time-dependent confounders are also affected by previous exposure, and g-methods are needed to properly adjust for time-varying confounding [30,32]. We applied a marginal structural model (i.e., a g-method) to properly adjust for time-varying confounding and possible selection bias due to differential loss to follow-up using baseline (age, gender, type of insurance, region, type of insurance product, history of opioid overdose, depression, alcohol use disorder, chronic pain, and CCI) and time-varying variables (alcohol use disorder, anti-depressants, benzodiazepines, methadone clinic visits, naltrexone, and MME). If the causal identification assumption holds, this method provides valid estimates of the effect of a time-varying OUD treatment on opioid overdose, while also accounting for time-varying confounding, which might be affected by OUD treatment history [43]. Most importantly, our study employed all available data sources and appropriate causal inference methods to best quantify the causal effect of receiving BUP-NX during follow-up on subsequent opioid overdose using routinely-collected data to address a question for which a randomized trial is no longer ethical or feasible.

We conducted a sensitivity analysis for the definition of lost to follow-up [16]. Suppose the outcome was from an external source, which is different from the source for exposure and other variables. In that case, it should be considered as ‘captured’ information and carried-forward until the end of the period corresponding to the loss-to-follow-up definition (six months in our study). In the sensitivity analysis, the average follow-up time was longer, and the number of events observed was larger than the main analysis due to the loss-to-follow-up definition. The point estimates of HRs from the MSMs for opioid overdose and composite outcomes were comparable between two different scenarios for the censoring mechanisms.

To conduct causal inference based on information from an observational study, the conditional exchangeability assumption must hold (i.e., given the measured baseline and time-varying variables, the exposure is independent of the counterfactual outcomes). This assumption cannot be tested with the observed data [15], however, sensitivity analyses can be conducted. We calculated the E-value for each outcome [44]. The E-value for the outcomes of opioid overdose, death, and composite endpoint were 2.4, 7.8, and 2.8, respectively. If there were any unmeasured confounders that violated the conditional exchangeability assumption and biased point estimate of the effect, the hazard ratio quantifying the association between the unmeasured confounder and exposure, or the unmeasured confounder and outcomes must be at least as strong as the E-values indicated above. We included many known risk factors for opioid overdose and death that were measurable in this database, and the calculated E-value was relatively large compared to the estimates for the relationship between measured covariates and the outcomes, especially for all-cause mortality. Based on this finding, unmeasured confounding may not completely explain away the observed effect of treatment on the endpoint of all-cause mortality. We note that establishing causality for the outcome mortality is challenging in this study because the outcome is not specific to OUD and there are likely important unmeasured confounders not captured in the administrative claims database.

This study has several limitations. Giving the nature of observational studies without a randomly assigned intervention, there could be unknown and/or unmeasured confounding. In our study, there is no information about OUD severity, illicit substance use, community naloxone distribution, and socioeconomic status variables (e.g., race, education level, income level), which could also be important confounders. The results also depend on the model being correctly specified. There could be measurement error using diagnosis codes in claims data [45]. The endpoint of opioid overdose alone fails to capture nonfatal opioid overdose events that happened outside of a hospital. To account for this concern, we defined a composite endpoint of opioid overdose or death, which could capture fatal/nonfatal opioid overdose in the hospital, fatal opioid overdose outside the hospital, and death due to other causes. The Death Master File does not capture all deaths, and actually records fewer death events than actually occurred; however, this would bias our result towards the null [46]. These study results could only be generalized to the target patient population included in this study, which was insured patients (at least 18 years old) diagnosed with opioid use disorder, who had not received buprenorphine, naltrexone, or methadone during the one-year period prior to study follow-up, who had at least 30-day of follow-up, and who were covered by commercial insurance or Medicare Advantage. The data source does not have enrollees of Medicaid or uninsured patients with OUD and these other groups represent distinct patient populations for which these results may not generalize.

In summary, we designed a retrospective cohort study using administrative claims data to evaluate buprenorphine-naloxone dispensing on health outcomes, including overdose. With an application of a marginal structural model under the conceptual trial emulation framework, our study provides evidence of the effectiveness of buprenorphine-naloxone treatment against opioid overdose and/or death. The findings of this study should be interpreted in light of its known limitations, including a retrospective design and possibly unmeasured important confounders and OUD disease severity. Future retrospective study with electronic health records that include more detailed information on confounders and prospective cohort studies will provide further insights into treatment of opioid use disorder.

## Supplementary Material

Supplemental File

## Figures and Tables

**Table 1.** The major protocol elements for a conceptual randomized trial studying the effect of buprenorphine-naloxone (BUP-NX) versus placebo and the design and method for the emulation retrospective cohort study using routinely-collected data.

Protocol Element	Conceptual Randomized Trial	Retrospective Cohort Study (Emulating the Conceptual Trial Using Routinely-Collected Data)
Eligibility criteria	Adults living with opioid use disorder (OUD) between 2010 and 2017 who have not received pharmacological treatment for OUD during the year before screening.	Same as the target trial. Use medical records and pharmacy claims to identify patients with OUD who have not received pharmacological treatment during the previous year. Require continuous enrollment for the screening year to ensure the continuity of the available information.
Treatment arms	Treatment arm: receiving buprenorphine-naloxone (BUP-NX) though the follow-up period; Control arm: placebo.	The active treatment is defined as the same as the treatment arm in the target trial. Placebo is unavailable in the claims data. Not receiving BUP-NX was defined as the comparison group. Use pharmacy claims to derive the BUP-NX dispensing information.
Treatment assignment procedures	After recruitment and screening, eligible patients are randomly assigned to either the treatment arm or the placebo arm.	Cannot perform randomization. Adjust for measured baseline and prognostic time-varying variables using statistical methods.
Follow-up period	The follow-up starts at the randomization. Patients are followed until endpoint occurs, loss-to-follow-up, or two years after the randomization, whichever happens first.	Without the milestone of randomization, the follow-up starts at the time when patients are first considered as eligible.
Endpoints	1. Opioid overdose; 2. All-cause mortality.	Same as the conceptual trial.
Causal parameters of interest	As-treated effects	Same as the conceptual trial.
Analysis Plan	Conduct statistical analysis using time-to-event methods. Estimate as-treated effect by comparing the hazard of endpoints using treatment adherence information adjusting for pre-specific baseline and prognostic variables associated with loss-to-follow-up and treatment adherence will be adjusted.	Use marginal structural models to estimate the effect of the time-varying treatment on endpoints adjusting for treatment dispensing, treatment history, measured baseline and time-varying confounders, and selection bias due to differential loss-to-follow-up.

**Table 2.** Descriptive summary of baseline demographic, medication, and comorbidity information of eligible and insured patients with opioid use disorder in the United States, 2010–2017.

Baseline Characteristics	Overall Cohort (*n* = 58,835)
Age (years) at beginning of follow-up, Mean (SD)	51 (17.6)
[IQR]	[37, 63]

Gender, n (%)	
Female	30,114 (51.2)
Male	28,721 (48.8)

Region, n (%)	
Midwest	10,615 (18.0)
Northeast	7361 (12.5)
South	26,267 (44.7)
West	14,592 (24.8)

Insurance type, n (%)	
Commercial	32,551 (55.33)
Medicare Advantage	26,284 (44.67)

Insurance product, *n* (%) *	
Exclusive provider organization	3749 (6.4)
Health maintenance organization	16,095 (27.4)
Point of service	24,639 (41.9)
Preferred provider organization	3484 (5.9)
Others	10,868 (18.5)

Medical conditions during baseline period, *n* (%)	
Chronic pain	46,559 (79.13)
Depression	27,847 (47.33)
Opioid overdose	2345 (3.99)
Alcohol use disorder	9011 (15.32)

CCI at baseline, Mean (SD)	1 (1.64)
[minimum, maximum]	[0, 12]

CCI = Charlson comorbidity index.

* Values of polytomous variables might not sum to 100% due to rounding.

**Table 3.** Total person-time and estimated hazard ratios (HRs) with 95% confidence intervals (CIs) of receiving BUP-NX dispensing versus not receiving BUP-NX on first opioid overdose, death, and a composite endpoint among eligible and insured patients with opioid use disorder in the United States, 2010–2017.

Parameter	Endpoint		

	First Opioid Overdose	Death	Composite Endpoint
Unadjusted model			
Total person-time, unit: person-year	63,910.6	65,180.2	63,881.6
Average follow-up time, unit: year	1.086	1.167	1.086
Number of events, *n* (%)	1908 (3.24)	1051 (1.79)	2874 (4.88)
Patients received at least one BUP-NX during follow-up, *n* (%)	5722 (9.73)	5835 (9.92)	5720 (9.72)
HR (95% CI)	0.71 (0.56, 0.88)	0.11 (0.05, 0.23)	0.50 (0.40, 0.62)

Marginal structural model			
Weighted total person-time, unit: person-year	64,906	66,897.5	65,183.76
Weighted follow-up time, Mean [Min, Max], unit: year	1.10	1.14	1.11
Weighted number of events, *n*	1904	1059	2874
HR (95% CI)	0.66 (0.49, 0.91)	0.24 (0.08, 0.75)	0.58 (0.40, 0.84)
Weight, Mean	1.02	1.03	1.02
E-value	2.4	7.8	2.8

## Data Availability

The data that support the findings of this study are available from Optum but restrictions apply to the availability of these data, which were used under license for the current study, and so are not publicly available. Data may be available upon reasonable request to Optum.
