# Supplementary material for: Evaluation of the Effectiveness of Buprenorphine-Naloxone on Opioid Overdose and Death among Insured Patients with Opioid Use Disorder in the United States
Source: Pharmacoepidemiology. Author manuscript; Available in PMC 2023 Feb 3. (PMC9896393; doi:10.3390/pharma1030010)

Supplemental Table S1. ICD-9/10-CM codes for opioid use disorder, opioid overdose, and other comorbidities

Condition	Version	Code
Opioid use disorder	9	304.00, 304.01, 304.02, 304.03, 304.70, 304.71, 304.72, 304.73, 305.50, 305.51, 305.52, 305.53
	10	F11, F192
Opioid overdose	9	965.00, 965.01, 965.02, 965.09, E85.00, E85.01, E85.02
	10	T40.0, T40.1, T40.2, T40.3, T40.6 (excluding T406.06 and T40.696)
Chronic pain	9	307.80, 307.89, 338.0, 338.2, 338.4, 719.41, 719.45-719.47, 719.49, 720.0, 720.2, 720.9, 721.0-721.4, 721.6, 721.8, 721.9, 722, 723.0, 723.1, 723.3-723.9, 724.0-724.6, 724.70, 724.79, 724.8, 724.9, 729.0-729.2, 729.4, 729.5
	10	F45.4, M08.1, M25.50, M25.51, M25.55 - M25.57, M43.2-M43.6, M45, M46.1, M46.3, M46.4, M46.9, M47, M48.0, M48.1, M48.8, M48.9, M50.8, M50.9, M51, M53.1-M53.3, M53.8, M53.9, M54, M60.8, M60.9, M63.3, M79.0-M79.2, M79.6, M79.7, M96.1
Depression	9	296.2, 296.3, 296.5, 300.4, 309, 311
	10	F20.4, F31.3–F31.5, F32, F33, F34.1, F41.2, F43.2
Alcohol misuse	9	265.2, 291.1–291.3, 291.5–291.9, 303.0, 303.9, 305.0, 357.5, 425.5, 535.3, 571.0–571.3, 980, V11.3
	10	E52, F10, G62.1, I42.6, K29.2, K70.0, K70.3, K70.9, T51, Z50.2, Z71.4, Z72.1

Supplemental Table S2. Results from sensitivity analysis with information being carried-forward for six additional 30-day intervals for those lost to follow-up among eligible and insured patients with opioid use disorder in the United States, 2010-2017

Parameter	Endpoint		
	First opioid overdose	Death	Composite endpoint
Unadjusted model			
Total person-time, unit: person- year	74812.7	76286.8	74713.8
Average follow-up time, unit: year	1.27	1.30	1.27
Number of events, n (%)	1962 (3.33)	1330 (2.26)	3191 (5.42)
HR (95% CI)	0.71 (0.57, 0.89)	0.17 (0.10, 0.29)	0.50 (0.41, 0.61)
Marginal structural model			
Weighted total person-time, unit: person-year	75417.98	77018.31	75360.20
Average weighted follow-up time, unit: year	1.28	1.31	1.28
Weighted number of events	1955	1318	3163
HR (95% CI)	0.73 (0.55, 0.96)	0.45 (0.22, 0.92)	0.69 (0.53, 0.90)

Supplemental Table S3. Estimated hazard ratios (HRs) with 95% confidence intervals (CIs) of measured baseline variables on each of the three outcomes among eligible and insured patients with opioid use disorder in the United States, 2010-2017

Baseline variables	Overdose<br>HR (95% CI)	Death<br>HR (95% CI)	Composite<br>HR (95% CI)
Age:<br>One year increase	0.979 (0.976, 0.983)	1.045 (1.039, 1.051)	0.998 (0.995, 1.001)
Gender:<br>Female versus male	1.002 (0.909, 1.105)	0.727 (0.639, 0.826)	0.912 (0.842, 0.989)
History of opioid overdose:<br>Yes versus no	2.932 (2.530, 3.397)	2.336 (1.902, 2.868)	2.712 (2.397, 3.070)
Region:<br>Midwest versus West	1.148 (0.995, 1.326)	0.831 (0.673, 1.025)	1.062 (0.936, 1.204)
Region:<br>Northeast versus West	0.966 (0.819, 1.139)	1.252 (1.021, 1.535)	1.045 (0.911, 1.199)
Region:<br>South versus West	0.805 (0.710, 0.912)	1.026 (0.882, 1.194)	0.858 (0.772, 0.954)
Chronic pain:<br>Yes versus no	0.934 (0.823, 1.059)	0.883 (0.718, 1.087)	0.832 (0.744, 0.930)
Depression:<br>Yes versus no	1.444 (1.302, 1.602)	1.211 (1.062, 1.380)	1.324 (1.217, 1.442)
Alcohol use disorder:<br>Yes versus no	0.986 (0.867, 1.121)	1.608 (1.356, 1.907)	1.131 (1.018, 1.257)
CCI:<br>One unit increase	1.218 (1.186, 1.251)	1.301 (1.264, 1.340)	1.259 (1.234, 1.285)

Supplemental Figure S1. Plot of adjusted cumulative incidence of opioid overdose versus time since opioid use disorder diagnosis (months) by time-varying Buprenorphine-Naloxone exposure status (yes versus no) among eligible and insured patients with opioid use disorder in the United States, 2010-2017

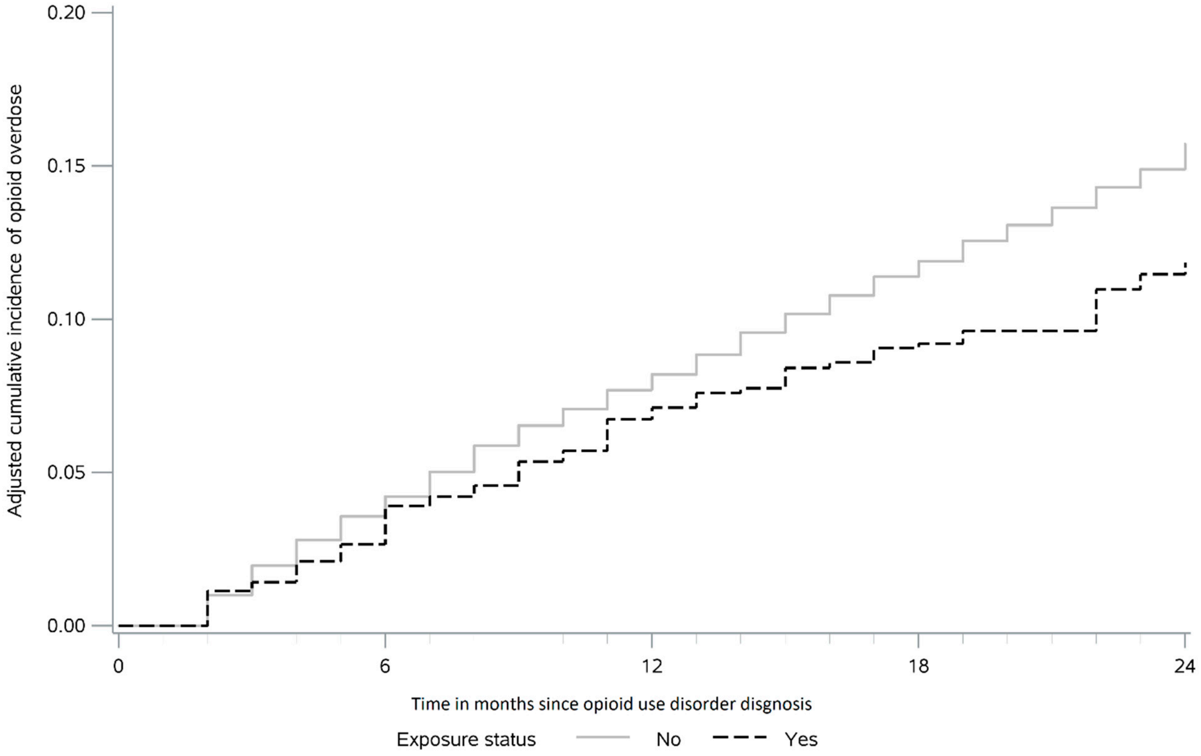

Supplemental Figure S2 Plot of adjusted cumulative incidence of all-cause mortality versus time since opioid use disorder diagnosis (months) by time-varying Buprenorphine-Naloxone exposure status (yes versus no) among eligible and insured patients with opioid use disorder in the United States, 2010-2017

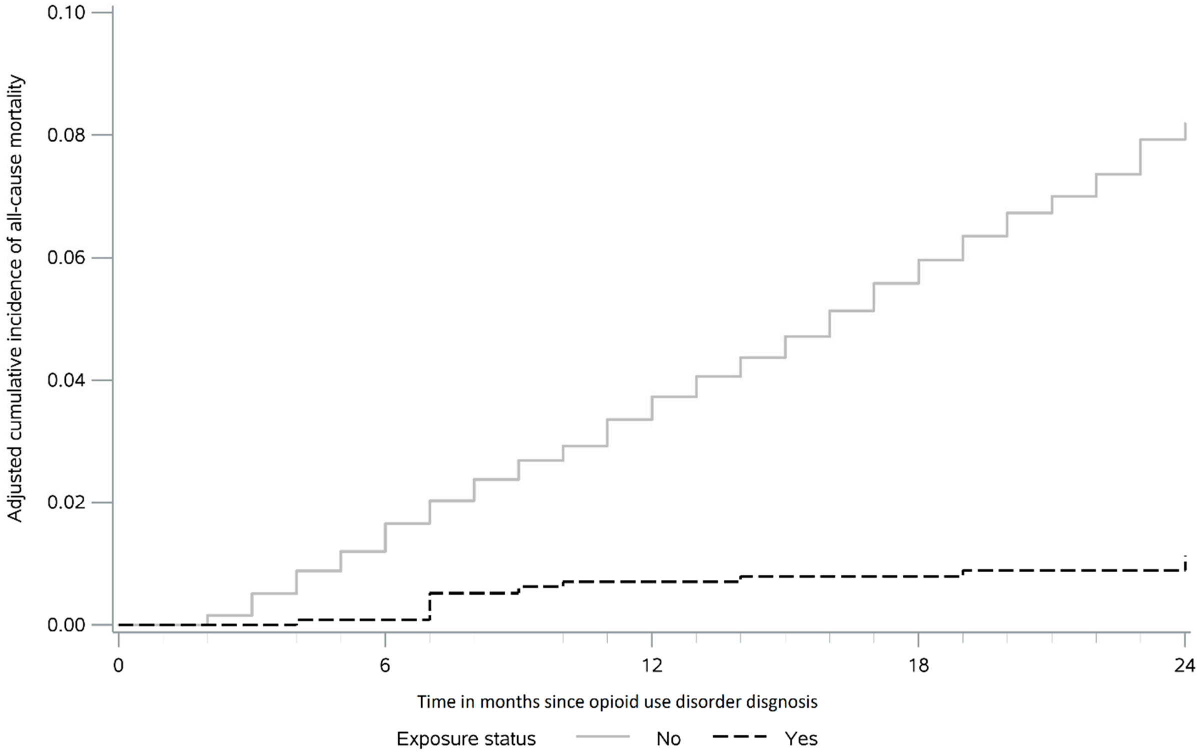

Supplemental Figure S3 Plot of adjusted cumulative incidence of composite endpoint of opioid overdose and all-cause death versus time since opioid use disorder diagnosis (months) by time-varying Buprenorphine-Naloxone exposure status (yes versus no) among eligible and insured patients with opioid use disorder in the United States, 2010-2017

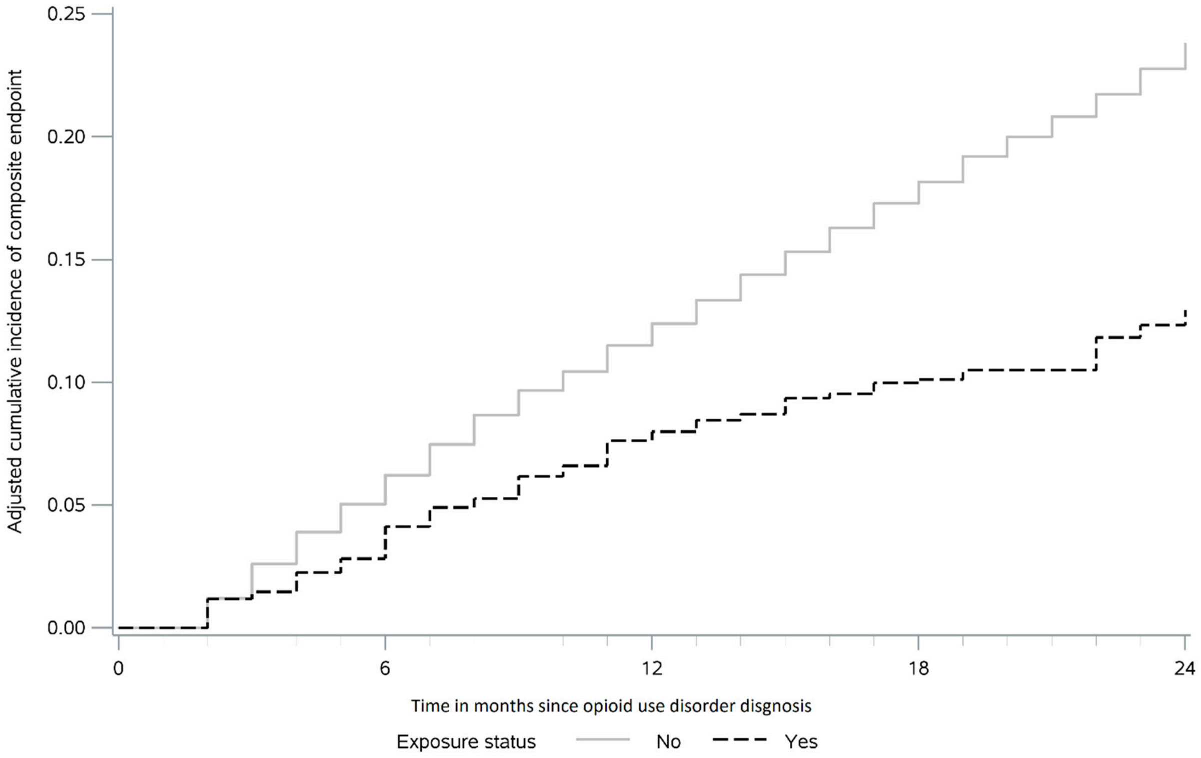

Supplement: Supplemental File [file NIHMS1854655-supplement-Supplemental_File.pdf]
